# Running faster. Standing still

**DOI:** 10.1002/nop2.80

**Published:** 2017-04-06

**Authors:** Philip Darbyshire

**Affiliations:** ^1^ Philip Darbyshire ConsultingAdelaideSouth AustraliaAustralia

Recently, I wondered if my and countless other nurses’ entire careers had been in vain. Forty plus years in clinical, education, research and culture change—and for what? What could provoke such angst? Read this new blog post from The King's Fund (2017a) and you will find out.

Chris Ham and Don Berwick may be the Lennon and McCartney of health policy and quality so when they speak, I definitely want to hear what's being said. Big hospitals, health services and NHS Trusts are fundamentally not working. They have a range of “key challenges” (for normal people, read “serious problems”). The King's Fund (2017b) has initiated a new research study called Frontline Clinical Care in Acute Hospitals to look into this, but their initial work suggests the following. This is the point where I realized that, like Toto, but in the year 2017, I was not in Kansas anymore either. This is their list of “challenges” that are holding back hospitals, damaging service quality and quite probably harming patients in the process. I am listing these as they do and insisting that you read every one. Do it now!

- Staff working under constant pressure (notwithstanding substantial increases in the number of clinical staff in recent years) in the face of growing demand from an ageing population with complex needs
- Difficulties for hospital staff in communicating with GPs about patients who are admitted to hospital, including knowing who patients’ GPs are
- Problems in communication within the hospital between acute medical staff and A&E staff, as well as between different specialist teams
- Difficulties in communicating with staff in other hospitals when patients are transferred
- Delays in ordering and receiving the results of diagnostic tests, which in turn lead to delays in treatment and increase in the time patients spend in hospital
- Challenges in teamworking, for example, on ward rounds when consultants are sometimes not accompanied by trainees and nurses
- Information systems that do not link data about patients held in primary and secondary care, and that are often slow in use
- Patients having to repeat their histories (where they are able to) at different stages in their treatment
- Care being delivered inefficiently and often ineffectively because of the amount of re‐work required by the above
- Old buildings and cramped layouts that do not allow privacy and sometimes dignity for patients, or space for staff to work without interruption
- Poorly organized paperwork and documents
- Inefficient organization of supplies and workflows on hospital wards.

Can you even begin to comprehend the enormity of what they are saying? Health professionals cannot, do not know how to, or do not want to communicate with each other. We do not share even the most basic information effectively. One ward or unit has really no idea what another does or how they should cooperate with each other. Different health professions and others involved in health care cannot or will not do this either. Hospitals do not integrate with any other hospitals or community services. Our essential documentation and recording is not even at rudimentary “fit for purpose” level. Our IT and computing systems are as bad or at an even worse level of functional uselessness.

Now that has sunk in, just imagine if you can, the countless billions—not millions—that have been spent over the last 40 years on quality initiatives, change programmes, reorganizations, management restructuring, strategic plans, leadership programmes, communication courses, consultancy fees, IT and electronic record roll‐outs, health professional education, “lean thinking” cures, health services research, regulatory processes, audit empires and more and more and more. Now think of all the people effort and career years involved. All of this money and effort involving good people, smart people, committed people and all for what?

What Ham and Berwick are describing as serious systemic problems in a major hospital that has been assessed as “outstanding”, look like health service and health professional 101 issues. What these would look like in a poorly performing hospital defies all description. This is not a complaint that we have not cured cancer yet. These are findings that we cannot communicate, that we cannot share vital information, that we cannot play nicely and cooperate in teams the way we would expect a group of kindergarten kids to, and that we do not know how to operate a connected, local system, let alone a truly national health service. Is it any wonder that a Parliamentary Report released in January this year was entitled *Will the NHS Never Learn?*’ (Public Administration and Constitutional Affairs Committee 2017). The parliamentarians’ frustrations were almost leaping off the pages. No wonder.

How would nurses react to such a damning assessment? Would we share the same views? Are we living in a similar *Groundhog Day* nightmare? Can we email Chris Ham and be confident that in nursing at least, we have solved most of these problems, and that we are not in such dire straits? Could we show tangible examples of exemplary teamwork, of seamless services, of stellar communication, of genuine integration and co‐operation that is stopping waste, improving lives and making a dramatic difference to people's health? For the sake of many of our careers and “legacies”, let alone the lives of our patients, clients and communities, I really hope so.
